# Purchases and prices of unprocessed or minimally processed foods according to food outlets and income in Brazil

**DOI:** 10.1017/S1368980026102481

**Published:** 2026-04-10

**Authors:** Daniela Silva Canella, Patricia Gálvez Espinoza, Ana Beatriz Coelho De Azevedo, Larissa Loures Mendes

**Affiliations:** 1 Institute of Nutrition, Rio de Janeiro State University, Brazil; 2 Department of Nutrition, Universidad de Chile, Chile; 3 Postgraduate Program in Food, Nutrition and Health, Rio de Janeiro State University, Brazil; 4 Departamento de Nutrição, Universidade Federal de Minas Gerais, Brazil

**Keywords:** Access to healthy foods, Food supply, Commerce, Affordability

## Abstract

**Objective:**

To assess the purchases and prices of unprocessed or minimally processed foods according to the type of food outlet and household income.

**Design:**

Cross-sectional study conducted with data from the 2017–2018 Brazilian Household Budget Survey. Food acquisition and income were the variables of interest. Unprocessed or minimally processed foods were identified according to the NOVA classification, and the shares of energy (kcal) and quantity (grams), as well as prices paid, were analysed. Food outlets were grouped into nine types. Household income per person was assessed in quintiles (Q). Descriptive analyses were conducted.

**Setting:**

Brazil.

**Participants:**

A nationally representative sample of 57 920 households.

**Results:**

The amount of unprocessed or minimally processed foods acquired varied from 320 g (Q1 of income) to 493 g (Q5). The increase in income had a positive effect on the share of foods purchased in supermarkets (Q1: 27·6 % v. Q5: 63·8 %) and fruit and vegetable retailers (Q1: 1·5 % v. Q4: 4·6 %). In contrast, an inverse relation was observed for Mini-markets (Q1: 34·9 % v. Q5: 16·2 %), butchers (Q1: 6·8 % v. Q5: 2·3 %), street markets (Q1: 13·3 % v. Q5: 3·8 %) and street food vendors (Q1: 5·3 % v. Q5: 1·0 %). The price paid for unprocessed or minimally processed foods in supermarkets, mini-markets, butchers and street markets was positively associated with income, which means that a higher mean price was observed in the highest income quintile.

**Conclusions:**

The availability and affordability of unprocessed or minimally processed foods differed according to food outlets and were influenced by income level.

Changes in dietary patterns, with an increase in the consumption of ultra-processed foods in replacement of unprocessed or minimally processed foods, have been observed in different populations worldwide^(1)^. In Brazil, the diet is still composed predominantly of unprocessed or minimally processed foods, which in 2017–2018 accounted for 48·7 % of the total energy purchased, whereas ultra-processed foods represented 19·4 %. However, dietary changes observed globally are also occurring in Brazil. Between 2002–2003 and 2017–2018, the energy share of unprocessed or minimally processed foods declined (by an average of 0·15 percentage points per year), whereas the share of ultra-processed foods increased (by an average of 0·31 percentage points per year)^(2)^. Unlike the consumption of unprocessed or minimally processed foods, which is recognised as protective against non-communicable diseases, the consumption of ultra-processed foods is associated with several adverse health outcomes^(3)^ and does not seem to contribute to a healthy, sustainable and fair food system^(1,4,5)^.

The change in the population’s diet has been explained by the characteristics of food environments where people engage in their daily activities^(6–8)^. Food availability and affordability are two relevant food environment variables or dimensions that have been highlighted in several frameworks^(4,9–13)^. Food availability refers to the presence, number, and types of food outlets or food retail as well as the availability of healthy foods within these outlets^(4,9–12)^. Food affordability, or economic access, is understood as a person’s capacity to pay for food and reflects the relative cost of food compared to an individual’s income or purchasing power^(4,9)^. A healthy food environment is characterised by the availability of healthy food in variety and at an affordable price^(14)^.

Among food availability research, evidence indicates that there are several outlet types where people can access food. Among these, supermarkets are claimed to play a significant role in food access because of the variety of available products and their prices, which can be more competitive than those of other stores. However, supermarkets also favor access to ultra-processed foods^(15,16)^.

Socioeconomic status is an essential aspect of food availability and affordability. Evidence from different countries shows that people from low socioeconomic backgrounds tend to be exposed to food environments with a smaller number of outlets that sell unprocessed and minimally processed foods^(17,18)^, and these foods are at higher prices^(19–21)^. For example, studies conducted in the United States and Australia showed that supermarkets are mainly importance in food access is observed mainly among populations living in more affluent areas or for high-income families. In contrast, low-income families tend to rely on smaller stores that have limited amounts of unprocessed food or are not of good quality^(22,23)^. Therefore, income can directly influence diet, affecting food purchases and access by determining the retailers available to individuals^(4)^. Consequently, people from low socioeconomic levels have a lower probability of reaching the recommended amount of fruit and vegetable intake^(24)^.

Given this context, the present study assessed the purchase and price of unprocessed or minimally processed foods by type of food outlet and household income in Brazil. Similar analyses have been conducted focusing specifically on fruits and vegetables but not on the entire category of unprocessed or minimally processed foods. Understanding healthy food access in different stores and by different income groups in low-and middle-income countries, such as Brazil, is fundamental to supporting the design, implementation, and improvements in public policies, such as the Brazilian National Food Supply Policy^(25)^, and effective interventions to promote healthy diets in territories, allowing people to reach adequate and healthy diets, based on unprocessed or minimally processed foods, according to the Brazilian Dietary Guidelines^(26)^.

## Methods

### Study population and data collection

We used data from the 2017–2018 Household Budget Survey (*Pesquisa de Orçamentos Familiares* – POF), the last edition of a representative survey of the Brazilian population, distributed among the country’s five regions, in urban and rural areas, and in all income groups, carried out by the Brazilian Institute of Geography and Statistics (*Instituto Brasileiro de Geografia e Estatística –* IBGE)^(27)^. The POF used a complex sampling plan by conglomerates, involving the geographic and socioeconomic stratification of all census tracts in the country, followed by the random selection of tracts in the first stage and households in the second stage. The sample consisted of 57 920 households grouped into 575 sampling strata. Data were collected between July 2017 and July 2018, ensuring that households were distributed evenly across the strata in the four trimesters to address the seasonality of expenditure and income^(27)^.

The basic information analysed in this study refers to household food availability and household income data. Food availability refers to food items purchased in each household for a period of seven consecutive days, recorded daily in a booklet provided by the IBGE. The booklet was completed by household residents or by an IBGE interviewer at the highest level of disaggregation available, along with the place where they were purchased, the quantity purchased and the price paid (R$)^(27)^. Due to the short reference period for recording food purchases in each household, sampling strata were used as the study unit, which are aggregates of households with geographical and socio-economic homogeneity, and whose annual food purchase patterns can be known with greater precision. In this study, non-monetary purchases (such as donations, business withdrawals, bartering, own production and others) were not considered because price was one of the attributes of interest.

Microdata of the POF is publicly available (https://www.ibge.gov.br/estatisticas/sociais/saude/24786-pesquisa-de-orcamentos-familiares-2.html). The information contained in the database does not allow participants to be identified, as specific data about each household, such as identification of household members, address and telephone are excluded.

### Creation and description of the variables

Regarding the food items purchased, the focus was on the healthy fraction of the diet through the group of unprocessed or minimally processed foods, defined according to the NOVA classification^(28)^, and the subgroup of fruits and vegetables.

The places where food was purchased were grouped based on an adaptation of the proposal by Costa *et al.* ^(15)^ and CAISAN^(29)^, considering the physical structure, nature and characteristics of the main products sold^(15,29)^. Nine groups of food outlets were established, as described in Table 1.

**Table 1.** Categorization of food outlets considering physical structure, type, and characteristics of the main products sold

Groups of food outlets	Types of establishments included
1. Supermarkets	Supermarkets, hypermarkets
2. Wholesalers	Wholesale hypermarkets, wholesale supermarkets, supply centres, bulk bottle water vendors/distributors, food distributors, grain merchants, food markets, among other wholesalers
3. Mini-markets	Mini-markets, grocery stores, public markets, municipal markets, small food stores, bars, emporiums, handmade food stores, frozen food stores
4. Butchers	Butchers, poultry houses, slaughterhouses, cold cuts stores, meat/chicken stores, meat processing companies, meat/fish markets, fishmongers, fish and pay, dairy shop/retailers, cheese shops
5. Fruit and vegetable retailers	Fruit/vegetable stand, fruit shop/stall, vegetable house, Cobal market, fruit shop, fruit and vegetable store, vegetable market, pay-per-kilo fruit and vegetable market, vegetable store, organic products store, vegetable gardens
6. Street markets	Street markets, street organic fruit and vegetable market, fruit/vegetable/fish/farming/agricultural product store, home-made product store, cooperative of farmers
7. Street food vendors	Street food vendors, stall, fruit truck, food trolley, street milkman/baker/fishmonger, food truck, kiosk/trailer, street assai vendor, etc.
8. Restaurants	Restaurants and the like; bars and the like; snack bars, tea parlours, juice stores and the like; bakeries and pastry shops, canteens-private food services; supply of food prepared for household consumption
9. Other	Sweets, candies, chocolates and the like; convenience stores; domestic production; establishments whose main purpose is not the sale of food, etc.

The parameters analysed for food items were energy, quantity and price. The total quantity of each food item purchased, expressed in kilograms, was transformed into the quantity purchased per day and per number of residents in the household. Correction factors were then applied to these data to exclude the inedible fraction of food and express the quantities available for consumption^(30)^, representing daily acquisition values (gram/per capita/day). The available quantity of each food was transformed into energy using the Brazilian Table of Food Composition from the University of São Paulo, Food Research Center, Version 7.0 (Available at: http://www.fcf.usp.br/tbca) to obtain the daily energy value acquired (kcal/per capita/day). The price paid for food items, in Brazilian Reais (R$), was used to calculate the price indicators^(31)^.

The following outcomes were constructed to be examined across different food outlet types: the relative energy share of unprocessed or minimally processed foods and fruits and vegetables purchased, as well as the relative quantity share of unprocessed or minimally processed foods, and fruits and vegetables. Additionally, regarding price indicators, the following outcomes were constructed to be examined across different food outlet types: the average price paid per energy (R$/1000 kcal)^(31)^ and the average price paid per quantity (R$/kg) of unprocessed or minimally processed foods and fruits and vegetables purchased.

Household income was also recorded in the survey. With this information and the number of dwellers in each household, the per capita household income was calculated (expressed in quintiles).

### Data analyses

The frequency of purchases reported at each type of food outlet, referring to the occurrence of purchases (i.e. if some food item, regardless of group or quantity, was acquired at that outlet), was described according to income quintiles and their respective 95 % confidence intervals (95 %CI).

The average energy share (%), average quantity share (%) and price indicators (R$/1000 kcal and R$/kg) of unprocessed or minimally processed foods were estimated for each of the nine food outlets groups. The same was estimated for the fruits and vegetables. All analyses were conducted for Brazil as a whole and according to the income quintiles. Averages of energy (kcal) and quantity (gram) of unprocessed or minimally processed foods and fruits and vegetables were also described.

Significant differences were identified by comparing the 95 % CI. The absence of overlap between the intervals was assumed to indicate a significant difference, considering a 5 % significance level.

The analyses were carried out in Stata/SE version 15.0 (Stata Corp.) in the survey module, so that the estimates considered the complexity of their sampling design and could be extrapolated to the Brazilian population.

## Results

In Brazil, supermarkets (51·42 %, 95 % CI 42·26, 60·48) were the food outlet most frequently referred for food purchases in general by the households, regardless of the food group or quantity of foods acquired, followed by restaurants (40·63 %, 95 % CI 32·56, 49·24) and mini-markets (31·64 %, 95 % CI 25·19, 38·87). However, the scenario differed considering the income level: among high-income households, 80·57 % (95 % CI 61·93, 91·36) reported purchases in supermarkets, and only 12·22 % (95 % CI 5·20, 26·13) and 2·36 (95 % CI 61·93, 91·36) in mini-markets and street markets, respectively; conversely, among low-income households, 47·52 % (95 % CI 38·07, 57·16) reported purchases in mini-markets, 22·57 % (95 % CI 16·36, 30·29) in supermarkets and 17·68 % (95 % CI 11·19, 26·81) in street markets (Table 2).

**Table 2.** Distribution of the frequency of reported purchases by food outlets^*^ according to income quintiles. Brazil, 2017–2018

			Quintiles of per capita household income									
	Brazil		Q1		Q2		Q3		Q4		Q5	
Food outlets	Relative frequency	95 % CI	Relative frequency	95 % CI	Relative frequency	95 % CI	Relative frequency	95 % CI	Relative frequency	95 % CI	Relative frequency	95 % CI
Supermarkets	51·42	42·26, 60·48	22·57	16·36, 30·29	46·75	32·42, 61·63	41·91	29·55, 55·37	66·17	50·48, 78·96	80·57	61·93, 91·36
Wholesalers	4·41	1·56, 11·81	3·75	1·48, 9·19	12·70	2·61, 44·18	0·92	0·21, 4·02	2·72	0·88, 8·03	1·24	0·19, 7·59
Mini-markets	31·64	25·19, 38·87	47·52	38·07, 57·16	34·01	22·87, 47·27	36·29	24·57, 49·90	30·42	18·89, 45·07	12·22	5·20, 26·13
Butchers	17·02	11·69, 24·10	19·06	11·64, 29·61	27·06	12·98, 47·99	7·02	2·97, 15·70	24·37	12·40, 42·32	8·94	3·22, 22·49
Fruit and vegetable retailers	5·34	3·40, 8·30	4·21	1·92, 8·99	3·82	1·42, 9·85	8·02	3·52, 17·24	9·91	3·94, 22·81	3·37	0·79, 13·24
Street markets	11·77	7·37, 18·29	17·68	11·19, 26·81	21·11	8·23, 44·39	5·94	2·30, 14·50	11·25	3·68, 29·64	2·36	0·70, 7·63
Street food vendors	8·63	5·70, 12·86	15·88	9·56, 25·20	4·83	2·44, 9·30	2·66	0·69, 9·74	11·15	3·69, 29·15	7·42	2·27, 21·69
Restaurants	40·63	32·56, 49·24	43·87	34·43, 53·77	48·77	34·57, 63·17	31·41	21·32, 43·62	45·33	30·32, 61·24	34·04	15·38, 59·43
Other^**^	8·59	5·89, 12·38	12·00	7·42, 18·85	6·13	2·76, 13·07	9·90	3·62, 24·31	10·88	4·05, 26·08	5·29	1·92, 13·74

* Refers to the occurrence of purchases in each kind of outlet, regardless of food group or quantity of foods acquired.

** Confectionery stores, pharmacies, convenience stores, stores where the main purpose is not to sell food and stores with generic specifications.

The daily per person energy and quantity of unprocessed or minimally processed foods purchased in Brazilian households varied from 669·78 kcal (95 % CI 628·24, 711·31) and 319·91 g (95 % CI 300·15, 369·68), in households with lower income (Q1), to 764·40 kcal (95 % CI 454·02, 1074·79) and 492·86 g (95 % CI 373·72, 612·00), in households with higher income (Q5) (Table 3). The values for fruits and vegetables varied significantly from 28·84 kcal (95 % CI 25·90, 31·77) and 55·58 g (95 % CI 50·13, 61·03), in households with lower income (Q1), to 50·61 kcal (95 % CI 35·84, 65·38) and 116·89 g (95 % CI 99·23, 134·55), in households with higher income (Q5) (Table 4).

**Table 3.** Distribution of energy (kcal) and quantity (grams) of unprocessed or minimally processed foods purchased by food outlet according to income quintiles. Brazil, 2017–2018

			Quintiles of per capita household income									
	Brazil		Q1		Q2		Q3		Q4		Q5	
Food outlets	Mean or Share	95 % CI	Mean or Share	95 % CI	Mean or Share	95 % CI	Mean or Share	95 % CI	Mean or Share	95 % CI	Mean or Share	95 % CI
Energy from unprocessed or minimally processed foods (kcal)	633·01	534·91, 731·11	669·78	628·24, 711·31	587·76	551·18, 624·34	532·24	481·87, 582·61	522·52	482·93, 562·11	764·40	454·02, 1074·79
Energy share of unprocessed or minimally processed foods (%)												
Supermarkets	51·32	47·15, 55·50	31·57	27·73, 35·42	47·68	42·33, 53·03	58·69	53·42, 63·46	58·69	52·64, 64·73	64·34	57·75, 70·93
Wholesalers	5·77	4·83, 6·71	6·82	4·71, 8·93	5·26	2·79, 7·73	4·76	3·00, 6·53	4·01	2·05, 5·98	6·85	5·11, 8·60
Mini-markets	28·16	25·75, 30·57	40·29	37·36, 43·22	29·81	25·22, 34·39	25·37	20·58, 30·16	24·92	19·75, 30·08	18·91	16·47, 21·34
Butchers	4·90	3·94, 5·87	6·36	5·63, 7·09	6·55	4·03, 9·08	3·96	3·07, 4·84	4·56	3·15, 5·98	2·93	1·26, 4·59
Fruit and vegetable retailers	1·49	1·14, 1·84	0·48	0·35, 0·60	1·00	0·57, 1·43	1·88	1·10, 2·65	2·20	1·56, 2·83	2·21	0·72, 3·69
Street markets	4·47	3·38, 5·56	8·43	6·38, 10·49	5·51	3·01, 8·02	2·47	1·42, 3·51	2·82	1·00, 4·63	2·05	0·67, 3·44
Street food vendors	1·34	1·08, 1·60	2·51	2·02, 3·00	1·56	1·04, 2·09	0·91	0·66, 1·17	0·78	0·54, 1·01	0·63	0·25, 1·02
Restaurants	1·36	1·11, 1·60	1·10	0·78, 1·42	1·68	1·16, 2·21	1·29	0·93, 1·64	1·15	0·84, 1·45	1·48	0·67, 2·29
Other^*^	1·20	0·94, 1·45	2·43	1·87, 2·99	0·93	0·61, 1·25	0·93	0·64, 1·22	0·88	0·60, 1·17	0·60	0·18, 1·01
Quantity from unprocessed or minimally processed foods (grams)	373·01	324·97, 421·04	319·91	300·15, 339·68	323·68	308·96, 338·40	346·32	319·22, 373·42	354·51	332·06, 376·97	492·86	373·72, 612·00
Quantity share of unprocessed or minimally processed foods (%)												
Supermarkets	48·40	43·21, 53·59	27·64	24·11, 31·16	44·03	39·44, 48·61	54·51	50·01, 59·00	55·45	49·97, 60·94	63·83	53·84, 73·81
Wholesalers	5·06	4·26, 5·86	5·58	3·93, 7·22	4·39	2·42, 6·37	4·86	2·78, 6·94	3·73	2·09, 5·38	6·02	4·59, 7·45
Mini-markets	25·10	22·56, 27·64	34·87	32·32, 37·42	27·26	23·04, 31·49	23·36	18·98, 27·64	23·31	18·98, 27·64	16·15	13·03, 19·27
Butchers	4·55	3·67, 5·43	6·84	6·09, 7·60	6·13	3·84, 8·42	3·31	2·64, 3·98	3·78	2·53, 5·02	2·29	1·10, 3·49
Fruit and vegetable retailers	3·40	2·68, 4·14	1·53	1·15, 1·92	2·81	1·68, 3·94	4·60	2·82, 6·38	4·56	3·24, 5·89	4·27	1·56, 6·98
Street markets	7·35	5·80, 8·90	13·34	10·67, 16·00	8·67	5·82, 11·53	4·46	2·64, 6·28	4·91	2·85, 6·97	3·81	1·27, 6·36
Street food vendors	2·48	1·98, 2·98	5·29	4·21, 6·37	2·45	1·97, 2·93	1·74	1·16, 2·32	1·30	0·93, 1·68	0·99	0·40, 1·58
Restaurants	2·16	1·83, 2·50	1·59	1·19, 2·00	2·91	2·11, 3·70	2·18	1·65, 2·70	1·98	1·42, 2·54	2·16	1·25, 3·06
Other^*^	1·49	1·18, 1·79	3·32	2·74, 3·90	1·35	0·97, 1·73	0·98	0·68, 1·28	0·97	0·61, 1·33	0·48	0·15, 0·82

* Confectionery stores, pharmacies, convenience stores, stores where the main purpose is not to sell food and stores with generic specifications.

**Table 4.** Distribution of energy and quantity of fruits and vegetables purchased by food outlet according to income quintiles. Brazil, 2017–2018

			Quintiles of per capita household income									
	Brazil		Q1		Q2		Q3		Q4		Q5	
Food outlets	Mean or Share	95 % CI	Mean or Share	95 % CI	Mean or Share	95 % CI	Mean or Share	95 % CI	Mean or Share	95 % CI	Mean or Share	95 % CI
Energy from fruits and vegetables (kcal)	40·20	37·41, 43·00	28·84	25·90, 31·77	37·53	33·37, 41·70	40·42	36·57, 44·27	44·59	41·58, 47·60	50·61	35·84, 65·38
Energy share of fruits and vegetables (%)												
Supermarkets	41·78	34·64, 48·91	18·23	14·46, 22·01	35·36	30·51, 40·21	49·02	43·90, 54·13	48·12	42·05, 54·20	61·22	45·52, 76·92
Wholesalers	3·26	2·67, 3·84	3·43	2·03, 4·83	2·84	1·59, 4·09	3·77	2·03, 5·51	2·88	1·64, 4·12	3·32	2·34, 4·30
Mini-markets	19·04	16·56, 21·52	21·98	18·92, 25·04	21·66	17·31, 26·01	19·31	14·76, 23·86	21·02	17·27, 24·78	12·74	8·28, 17·21
Butchers	0·20	0·8, 0·32	0·34	0·07, 0·61	0·39	–0·05, 0·83	0·11	–0·00, 0·83	0·08	0·03, 0·12	0·04	–0·00, 0·08
Fruit and vegetable retailers	10·67	8·99, 12·36	7·38	5·60, 9·17	10·62	6·63, 14·62	13·25	8·57, 17·92	13·11	9·25, 16·98	10·78	6·58, 14·97
Street markets	19·12	15·32, 22·92	37·86	31·82, 43·90	20·66	15·44, 25·88	11·31	6·30, 16·32	11·30	7·26, 15·33	9·56	3·09, 16·03
Street food vendors	3·88	2·94, 4·82	7·56	5·66, 9·46	4·61	2·56, 6·66	2·37	1·41, 3·32	2·45	1·45, 3·45	1·56	0·23, 2·89
Restaurants	0·93	0·34, 1·53	0·98	0·11, 11·84	2·31	–0·00, 4·61	0·37	0·19, 0·22	0·15	0·07, 0·22	0·55	0·05, 1·05
Other^*^	1·12	0·67, 1·57	2·24	1·46, 3·03	1·55	0·00, 3·10	0·49	0·16, 0·81	0·89	0·04, 1·74	0·23	0·00, 0·46
Quantity from fruits and vegetables (grams)	84·27	80·07, 88·47	55·58	50·13, 61·03	73·20	66·19, 80·22	82·89	74·92, 90·85	92·85	86·39, 99·32	116·89	99·23, 134·55
Quantity share of fruits and vegetables (%)												
Supermarkets	42·68	34·86, 50·50	19·24	15·35, 23·13	36·92	32·60, 41·24	48·60	43·43, 53·77	47·87	41·91, 53·83	63·00	44·84, 81·16
Wholesalers	3·44	2·64, 4·23	3·57	2·19, 4·95	3·23	1·49, 4·96	4·45	1·95, 6·95	3·04	1·66, 4·42	3·05	1·56, 4·54
Mini-markets	18·47	15·80, 21·14	21·98	19·04, 24·92	21·16	17·16, 25·15	18·53	14·53, 22·53	20·16	16·35, 23·97	11·87	6·47, 17·27
Butchers	0·21	0·10, 0·32	0·27	0·13, 0·41	0·48	–0·01, 0·97	0·12	0·02, 0·22	0·10	0·03, 0·17	0·05	0·01, 0·08
Fruit and vegetable retailers	10·80	8·91, 12·68	7·66	5·81, 9·52	10·69	6·76, 14·63	13·94	9·17, 18·71	13·28	9·27, 17·30	10·40	5·31, 15·50
Street markets	18·92	15·20, 22·63	37·40	31·44, 43·37	19·93	15·17, 24·68	11·48	6·87, 16·09	11·94	7·79, 16·09	9·32	3·08, 15·55
Street food vendors	3·66	2·81, 4·51	7·07	5·24, 8·90	4·18	2·55, 5·80	2·15	1·38, 2·92	2·51	1·50, 3·53	1·63	0·36, 2·89
Restaurants	0·83	0·29, 1·36	0·82	0·06, 1·58	2·09	–0·02, 4·19	0·32	0·17, 0·47	0·18	0·08, 0·27	0·46	0·05, 0·88
Other^*^	1·00	0·62, 1·38	1·99	1·29, 2·68	1·33	0·07, 2·60	0·42	0·16, 0·67	0·91	0·19, 1·63	0·23	0·01, 0·44

* Confectionery stores, pharmacies, convenience stores, stores where the main purpose is not to sell food and stores with generic specifications.

Income increase had a direct effect on the quantity share of unprocessed or minimally processed foods purchased in supermarkets (27·64 % in Q1 *v*. 64·34 % in Q5) and fruit and vegetable (FV) retailers (1·53 % in Q1 and 4·56 % in Q4) and on the energy share purchased in supermarkets (31·57 % in Q1 *v*. 63·83 % in Q5) and FV retailers (0·48 % in Q1 and 2·21 % in Q5). However, an inverse relationship was observed for mini-markets: the energy and quantity shares purchased varied from 40·29 % and 34·87 % in Q1 to 18·91 % and 16·15 % in Q5, respectively. Important variations in the same direction were also observed in street markets, butchers and street food vendors; low-income households had a higher share of purchases at these places (Table 3). Considering fruits and vegetables, similar results related to availability were observed: income had a direct relationship with purchases in supermarkets and an inverse relationship with acquisitions in minimarkets, street markets and street food vendors, despite this last outlet contributing to a small share of the purchases (Table 4).

The mean price per energy (R$/1000 kcal) of unprocessed or minimally processed foods was lower in mini-markets (3·80, 95 % CI 3·54, 4·06), supermarkets (3·92, 95 % CI 3·72, 4·13) and wholesalers (4·13, 95 % CI 3·56, 4·70); intermediate in restaurants (6·98, 95 % CI 5·26, 8·71), street food vendors (7·41, 95 % CI 6·47, 8·35) and street markets (8·56, 95 % CI 7·51, 9·62) and higher in FV retailers (11·34, 95 % CI 8·91, 13·76) and butchers (10·29, 95 % CI 9·51, 11·07). The prices paid at supermarkets, mini-markets, butchers and street markets were positively associated with income. However, when considering the mean price per quantity (R$/kg), except for butchers (17·60, 95 % CI 16·76, 18·43), the mean price was around R$ 6·00/kg for wholesalers, street markets, restaurants, mini-markets, FV retailers, supermarkets and street food vendors. Only the price paid at butchers increased with income (Table 5).

**Table 5.** Distribution of the mean price (R$/1000 kcal and R$/kg) of unprocessed or minimally processed foods purchased by food outlets according to income quintiles. Brazil, 2017–2018

			Quintiles of per capita household income									
	Brazil		Q1		Q2		Q3		Q4		Q5	
Food outlets	Mean price^**^	95 % CI	Mean price^**^	95 % CI	Mean price^**^	95 % CI	Mean price^**^	95 % CI	Mean price^**^	95 % CI	Mean price^**^	95 % CI
R$/1000 kcal												
Supermarkets	3·92	3·72, 4·13	3·06	2·71, 3·41	3·54	3·04, 4·04	3·78	3·52, 4·03	4·31	3·90, 4·72	5·00	4·58, 5·42
Wholesalers	4·13	3·56, 4·70	2·88	1·87, 3·90	3·41	2·66, 4·16	5·09	3·96, 6·23	4·88	3·59, 6·17	4·64	3·27, 6·01
Mini-markets	3·80	3·54, 4·06	2·76	2·53, 3·00	3·29	3·00, 3·57	3·84	3·54, 4·15	4·25	3·79, 4·71	4·97	4·35, 5·59
Butchers	10·29	9·51, 11·07	8·58	7·96, 9·21	8·17	7·54, 8·81	10·54	8·40, 12·68	11·70	10·05, 13·36	12·86	11·12, 14·59
Fruit and vegetable retailers	11·34	8·91, 13·76	9·88	8·54, 11·22	8·89	7·22, 10·56	16·04	5·41, 26·68	9·67	8·06, 11·27	12·77	10·51, 15·03
Street markets	8·56	7·51, 9·62	6·46	5·79, 7·13	6·87	6·05, 7·69	9·11	7·45, 10·77	10·78	5·95, 15·62	10·09	8·81, 11·37
Street food vendors	7·41	6·47, 8·35	6·03	5·04, 7·03	8·81	6·11, 11·50	6·47	5·22, 7·73	7·70	6·37, 9·03	7·83	5·82, 9·84
Restaurants	6·98	5·26, 8·71	5·86	5·18, 6·54	6·72	5·93, 7·52	5·00	4·31, 5·69	10·10	0·89, 19·32	7·40	5·83, 8·97
Other^*^	19·09	–1·13, 39·32	5·80	4·16, 7·44	7·07	5·47, 8·67	10·06	4·81, 15·31	67·54	–43·33, 178·41	10·68	6·15, 15·21
R$/kg												
Supermarkets	6·66	6·39, 6·93	6·47	6·01, 6·94	7·08	6·43, 7·72	5·99	5·60, 6·38	6·71	6·15, 7·27	6·95	6·66, 7·24
Wholesalers	6·19	5·65, 6·72	5·55	4·39, 6·70	6·51	5·53, 7·48	6·06	4·66, 7·45	6·35	5·10, 7·59	6·46	5·27, 7·64
Mini-markets	6·40	6·20, 6·61	6·19	5·83, 6·55	6·58	6·26, 6·89	6·02	5·63, 6·42	6·31	5·83, 6·79	6·85	6·39, 7·31
Butchers	17·60	16·76, 18·43	15·87	15·04, 16·70	14·80	13·86, 15·74	17·83	15·89, 19·77	19·17	17·31, 21·04	20·80	19·01, 22·59
Fruit and vegetable retailers	6·48	5·84, 7·10	5·95	5·12, 6·78	5·17	4·70, 5·64	6·34	5·05, 7·63	7·35	5·31, 9·38	7·76	6·53, 9·00
Street markets	6·31	5·87, 6·73	6·15	5·61, 6·69	7·41	6·34, 8·48	5·76	5·35, 6·16	5·80	5·24, 6·36	6·18	5·43, 6·93
Street food vendors	6·77	6·10, 7·44	6·24	4·95, 7·52	7·04	5·73, 8·36	6·44	3·94, 8·94	7·46	6·53, 8·39	6·70	5·82, 7·57
Restaurants	6·37	5·69, 7·06	6·97	5·62, 8·33	7·71	6·55, 8·87	4·39	3·43, 5·35	5·06	4·09, 6·03	7·24	5·72, 8·76
Other^*^	13·60	9·00, 18·21	7·02	5·80, 8·24	9·03	6·65, 11·40	22·98	3·29, 42·99	16·34	9·69, 22·99	14·44	10·28, 18·59

* Confectionery stores, pharmacies, convenience stores, stores where the main purpose is not to sell food and stores with generic specifications.

** R$ 1·00 = US$ 0·18 (July 11, 2024).

For fruits and vegetables, the mean price (R$/1000 kcal and R$/kg) was lower in mini-markets (9·98, 95 % CI 9·10, 10·85 and 5·38, 95 % CI 5·12, 5·64, respectively); however, it did not vary much among the food outlets, except for butchers, which had a higher price, and in each outlet across the income quintiles (Table 6).

**Table 6.** Distribution of the mean price (R$/1000 kcal and R$/kg) of fruits and vegetables purchased by food outlets according to income quintiles. Brazil, 2017–2018

			Quintiles of per capita household income									
	Brazil		Q1		Q2		Q3		Q4		Q5	
Food outlets	Mean price^**^	95 % CI	Mean price^**^	95 % CI	Mean price^**^	95 % CI	Mean price^**^	95 % CI	Mean price^**^	95 % CI	Mean price^**^	95 % CI
R$/1000 kcal												
Supermarkets	12·66	11·10, 14·21	11·91	10·98, 12·84	11·52	10·23, 12·80	13·66	11·66, 15·65	14·76	11·05, 18·47	10·97	9·99, 11·95
Wholesalers	11·97	10·49, 13·46	12·80	10·53, 15·07	13·37	11·07, 15·67	11·58	7·25, 15·91	9·75	8·62, 10·88	11·39	9·65, 13·13
Mini-markets	9·98	9·10, 10·85	10·78	9·84, 11·72	9·71	9·33, 10·09	11·16	5·98, 16·35	9·11	7·14, 11·08	9·55	6·46, 12·64
Butchers	29·94	15·70, 44·18	40·17	7·05, 73·30	12·54	10·17, 14·90	21·83	6·87, 36·80	40·62	19·23, 62·00	21·30	13·04, 29·56
Fruit and vegetable retailers	12·00	9·38, 14·62	11·65	10·19, 13·12	13·48	6·90, 20·07	14·97	0·44, 29·50	10·33	7·92, 12·73	10·18	5·10, 15·26
Street markets	10·40	8·30, 11·57	9·65	8·96, 10·35	9·86	8·75, 10·97	10·24	6·98, 13·50	11·84	11·31, 12·36	10·52	8·34, 12·70
Street food vendors	9·94	8·30, 11·57	9·76	8·10, 11·41	9·23	6·28, 12·19	10·75	7·94, 13·57	11·01	6·73, 15·29	8·00	2·12, 13·89
Restaurants	16·23	11·13, 21·32	13·73	9·86, 17·60	18·70	7·20, 30·20	13·65	9·99, 17·30	17·31	3·69, 30·93	15·17	15·01, 15·34
Other^*^	17·65	12·10, 23·20	14·37	8·75, 20·00	17·63	12·94, 22·32	39·03	–1·95, 80·00	17·17	3·58, 30·75	9·09	9·04, 9·14
R$/kg												
Supermarkets	6·07	5·42, 6·73	5·61	5·26, 5·95	5·78	5·19, 6·38	6·56	6·04, 7·08	6·92	5·20, 8·63	5·36	5·03, 5·70
Wholesalers	5·75	5·24, 6·26	5·96	5·10, 6·83	5·87	5·43, 6·32	5·11	3·92, 6·30	5·60	4·18, 7·01	5·45	5·00, 5·90
Mini-markets	5·38	5·12, 5·64	5·38	5·06, 5·71	5·16	4·81, 5·51	5·33	3·71, 6·94	5·66	5·36, 5·97	5·23	5·09, 5·38
Butchers	9·49	5·16, 13·82	12·89	1·93, 23·85	4·60	3·57, 5·63	8·52	5·65, 11·39	10·33	3·63, 17·03	13·96	3·28, 24·63
Fruit and vegetable retailers	6·63	5·10, 7·69	5·99	4·95, 7·03	6·53	4·64, 8·42	7·27	4·90, 9·64	7·60	5·57, 9·62	4·87	4·49, 5·25
Street markets	5·50	5·13, 5·86	5·60	4·87, 6·34	5·24	4·71, 5·76	4·69	4·20, 5·18	5·96	5·59, 6·32	4·97	4·49, 5·46
Street food vendors	6·39	5·10, 7·69	6·90	5·94, 7·85	6·24	4·31, 8·16	5·18	4·47, 5·90	6·52	2·51, 10·53	5·22	4·32, 6·12
Restaurants	8·89	6·06, 11·72	11·24	4·84, 17·64	9·59	6·38, 12·79	8·37	5·48, 11·26	4·91	2·63, 7·19	12·81	8·55, 17·07
Other^*^	13·12	5·09, 21·15	11·23	3·21, 19·24	23·53	–2·39, 49·44	8·20	6·05, 10·36	6·64	3·17, 10·12	6·36	5·52, 7·20

* Confectionery stores, pharmacies, convenience stores, stores where the main purpose is not to sell food and stores with generic specifications.

** R$ 1·00 = US$ 0·18 (July 11, 2024).

## Discussion

Using nationally representative data from households in a middle-income country, this study examined the effect of household income on access to unprocessed or minimally processed foods, focusing on purchases made at various food outlets and their relation to prices paid. Generally, supermarkets and mini-markets were the primary sources of unprocessed or minimally processed foods, with street markets playing a significant role in fruit and vegetable purchases. However, the trends varied with income levels: the proportion of purchases made in supermarkets increased with income, while spending at mini-markets, street markets, butchers and street food vendors decreased.

Regarding the price of healthy foods, differences were noted across food groups studied when considering the price per energy and price per quantity. For unprocessed or minimally processed foods, the average price per quantity remained relatively stable across different food outlets and income levels. Conversely, the average price per energy was lower in mini-markets, supermarkets and wholesalers, but tended to increase with income. Fruits and vegetables exhibited a lower average price in mini-markets; however, this price varied across different food outlets based on the income levels.

Considering our results, three types of food outlets deserve attention: supermarkets and mini-markets for unprocessed or minimally processed foods in general and street markets for fruits and vegetables. The Pan American Health Organization classifies supermarkets as modern food retail outlets, whereas mini-markets and street markets are traditional food retail outlets^(1)^. Interestingly, the results revealed that modern retail was responsible for a higher share of purchases among high-income households in Brazil, whereas traditional retail was more accessible by low-income families. This could be related to the lower prices and locations of these traditional retails in some contexts^(32)^.

Globally, between 2009 and 2023, the retail food environment experienced an increase in the density of chain outlets (such as supermarkets and hypermarkets), and unhealthy food sales per capita were observed, whereas non-chain outlet density declined^(33)^. Although supermarkets contribute to access to healthy food in Brazil, mainly for more affluent households, as verified in our study, they can also be considered a food swamp (which refers to neighbourhoods with high availability of unhealthy foods)^(34)^ because of their contribution to ultra-processed food access^(1,15,16,35,36)^. Furthermore, although supermarkets contribute to access to unprocessed or minimally processed foods, they can have a drastic impact on other food outlets with economically unfair competition^(4)^ against mini-markets, FV retailers and street markets, which play an important role in access to healthy foods. In Mexico, another middle-income country, the density of convenience stores, discount stores, supermarkets (all modern retail) and small grocery stores (traditional retail) grew between 2010 and 2018, while the density of stores specialised in fruit/vegetables/seeds kept stable^(36)^. Interventions, such as zoning regulations to restrict the presence of supermarkets, along with support to traditional retailers, such as street markets, can contribute to improving the quality of food environments^(4)^.

Direct comparisons with other food outlets across the world are not easy because of the plurality of retail outlets, their characteristics and their role in food access in diverse contexts, such as small cities *v*. metropolises, rural *v*. urban areas, more affluent *v*. less affluent areas and high-income *v*. low-or middle-income countries.

Mini-markets seem to be important for guaranteeing healthy food access in Brazil, especially for low-income families. This could be related to the variety and price of food items sold there; however, another hypothesis for this phenomenon is their location. They tend to be present in small cities, rural areas and outskirts, making physical access easier for vulnerable populations^(37–39)^. However, to some extent, mini-markets are similar to supermarkets in terms of access to healthy and unhealthy food^(40,41)^, which can stigmatise them as a source of ultra-processed food. In this context, interventions to support managers and retailers in promoting healthy food environments are relevant. These interventions can include changes in establishments designed to incentivise healthy choices, such as displaying fresh food in the cashier area and at the ends of gondolas, distributing materials, producing social media content and adding labels to identify healthy foods^(42,43)^.

Additionally, food supply policies should pay attention to these outlets and provide incentives to improve the availability of unprocessed or minimally processed foods as a way of valuing local commerce. Strategies to improve healthy food access, focusing on more vulnerable areas, are important because, in Mexico, for example, despite the food environment continuing to be mostly represented by traditional retailers, between 2010 and 2020, a substantial expansion of convenience stores and supermarkets in more deprived and less urbanised areas was observed^(44)^.

Street markets, also referred to as open-air markets or wet markets, are traditional places for the acquisition of unprocessed or minimally processed foods in low-and middle-income countries^(10,45)^, and their availability close to households is associated with fruits and vegetable consumption^(46,47)^. Although in the biggest Brazilian metropolis, street markets tend to be more concentrated in high-income areas^(47)^, which contradicts our findings of greater access among low-income families, they are nonetheless distributed across all regions of the country and are available in 66 % of municipalities^(48)^. According to our results, the mean price paid for fruits and vegetables in street markets was not high compared to other outlets. Street markets can be considered territorial markets and are spaces where relationships are built because they allow the establishment of a connection between consumers and farmers, which is relevant for promoting a healthy food system^(45)^.

Regarding prices, the mean values paid for unprocessed or minimally processed foods were relatively stable across income levels, but the price for energy differed for food outlets, with supermarkets and mini-markets presenting lower values. This probably reflects the greater variety of items available in these outlets. These Brazilian results differ from those in the USA, where smaller food outlets presented higher prices for most staple foods than their closest supermarkets^(32)^. However, for fruits and vegetables, the values were more similar, except for butchers, even when comparing modern and traditional food outlets.

In addition to food supply policies, we believe that our results are also relevant in the context of programmes that deliver food vouchers to individuals. The Brazilian Workers’ Food Program was established in 1976 under Law No. 6·321, with voluntary adherence by companies based on fiscal incentives under one or more modalities (offering meals at the workplace, giving meal vouchers, food vouchers or food baskets). It aims to improve the nutritional status of workers, mainly low-income workers, to promote their health and prevent occupational diseases. In 2022, it covered approximately 24 million workers, 40 % of whom received food vouchers. Currently, there are no specifications of food items or outlets that workers can use vouchers, which can contribute to unhealthy practices^(49)^. In the UK, in the Healthy Start, a program of the National Health Service, women more than 10 weeks pregnant and children under 4 years may be eligible to receive a card to buy healthy foods such as fruits, vegetables, pulses and milk in some shops^(50)^. Recognising the contribution of traditional outlets, such as mini-markets and street markets, to the purchase of unprocessed or minimally processed foods by low-income families can stimulate public actions directed to these outlets.

This study has some limitations. The data were collected prior to the COVID-19 pandemic, during a period marked by neoliberal governments that showed limited commitment to social issues (2016–2022), followed by a recovery in the state’s role in ensuring food security^(25)^. Therefore, the current scenario may differ. Data on restaurants are underestimated because they refer only to the acquisition of food for consumption at home. Furthermore, it is necessary to highlight that the nature of food purchased in restaurants differs from that bought in other food outlets, which can affect their evaluation and characteristics. Additionally, outlets that offer a more limited range of food items, such as butcher shops and fruit and vegetable retailers, may provide restricted estimates of the prices paid for unprocessed or minimally processed food.

The strengths of this study are highlighted. Food purchase data were obtained from the last edition of a nationally representative survey covering all regions and income levels, disaggregated for different food outlets, which allowed us to explore the role of the types of food outlets in healthy food access. Energy and quantity parameters were combined to analyse the purchase of unprocessed or minimally processed foods, as well as fruits and vegetables. This combination is believed to enable a more comprehensive analysis of food purchases across different food outlet types, surpassing the traditional focus on energy availability, which is not always sufficient or useful for policy formulation and understanding supply dynamics. In addition, the use of two price indicators is interesting because the price per energy tends to be used more, but the price per quantity could be better for planning supply actions. Additionally, we used the NOVA food classification, adopted by the Brazilian Dietary Guidelines^(26)^, to define healthy food and assess food acquisition, contributing to the comprehension and overcoming of two of the six obstacles to achieving the Guideline’s recommendations: supply and cost.

### Conclusions

Household income influences the choice or access to food retail for the purchase of healthy food in Brazil: supermarkets were responsible for a higher share of purchases among high-income households in the country, while mini-markets, butchers and street markets were more accessible to Brazilian low-income families. In Brazil, the availability and affordability of unprocessed or minimally processed foods in different food outlets are influenced by income. Studies on the food environment have mainly focused on food availability, but understanding the effect of income on access to different food retailers is relevant for designing food supply interventions. These findings underscore the importance of considering income as a critical factor in food access and dietary choices, particularly when designing policies aimed at improving nutrition among vulnerable populations in low-and middle-income countries.
